# New elephant crisis in Asia—Early warning signs from Myanmar

**DOI:** 10.1371/journal.pone.0194113

**Published:** 2018-03-13

**Authors:** Christie Sampson, John McEvoy, Zaw Min Oo, Aung Myo Chit, Aung Nyein Chan, David Tonkyn, Paing Soe, Melissa Songer, A. Christy Williams, Klaus Reisinger, George Wittemyer, Peter Leimgruber

**Affiliations:** 1 Department of Biological Sciences, Clemson University, Clemson, SC, United States of America; 2 Smithsonian Conservation Biology Institute, National Zoological Park, Front Royal, VA, United States of America; 3 Myanma Timber Enterprise, Ministry of Natural Resources and Environmental Conservation, Yangon, Myanmar; 4 Department of Fish, Wildlife, and Conservation Biology, Colorado State University, Fort Collins, CO, United States of America; 5 WWF–Myanmar, Yangon, Myanmar; 6 Department of Biology, University of Arkansas at Little Rock, Little Rock, AR, United States of America; 7 Compass Films, Vincennes, France; University of Kwazulu-Natal, SOUTH AFRICA

## Abstract

In the southern Bago Yoma mountain range in Myanmar, Asian elephants are being killed at a disturbing rate. This emerging crisis was identified initially through a telemetry study when 7 of 19 of collared elephants were poached within a year of being fitted with a satellite-GPS collar. Subsequent follow up of ground teams confirmed the human caused death or disappearance of at least 19 elephants, including the seven collared individuals, within a 35 km^2^ area in less than two years. The carcasses of 40 additional elephants were found in areas located across south-central Myanmar once systematic surveys began by our team and collaborators. In addition to the extreme rate of loss, this study documents the targeting of elephants for their skin instead of the more common ivory, an increasing trend in Myanmar. Intensive research programs focused on other conservation problems identified this issue and are now encouraging local authorities to prioritize anti-poaching efforts and improve conservation policies within the country. Myanmar represents one of the last remaining countries in Asia with substantial wildlands suitable for elephants. Increasing rates of human-elephant conflict and poaching events in this country pose a dire threat to the global population.

## Introduction

The poaching crisis for African elephants (*Loxodonta africana*, B.), driven by the illegal ivory trade, has been well documented and receives much international attention in the media and scientific literature [1, 2, 3, 4, 5, 6]. Poaching of Asian elephants (*Elephas maximus*, L.) for ivory has been a major threat to the species [7], though because only male Asian elephants have tusks and proportions of tusked males varies between 10% -90% in different areas [7,8], this form of mortality has affected some populations more than others. Additional conservation concerns are focused on the impacts of live-captures for temples as well as for logging and tourist camps [9], and escalating human-elephant conflict. Local governments generally see human-elephant conflict as the primary threat to elephant populations throughout many parts of their range, probably because this type of threat is visible and affects the health and welfare of human populations. Incidental reports have indicated that elephants are poached for skin, meat, genitalia, and hair on occasion, but the demographic impacts from this trade were thought to be minor relative to those from the ivory and live trade [10].

**Fig 1:**
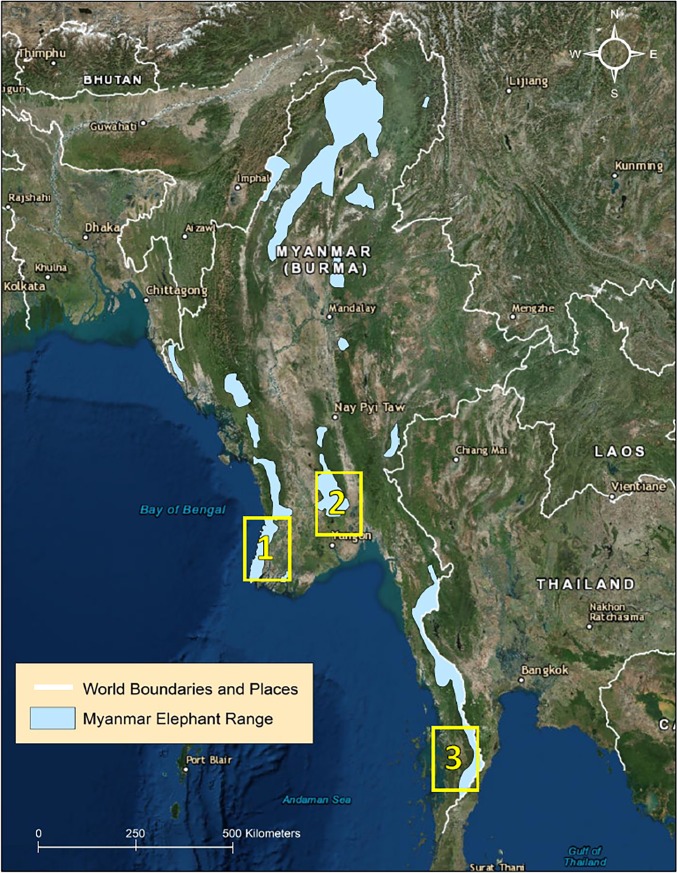
Locations of the study sites in Myanmar. 1) Ayeryarwady Delta, 2) southern Bago Yoma mountain range, and 3) southern Tanintharyi region. Myanmar elephant range from [18]. Image source: World Imagery: Esri, DigitalGlobe, Earthstar Geographics, CNES/Airbus DS, GeoEye, USDA FSA, USGS, Getmapping, Aerogrid, IGN, IGP, and the GIS User Community (accessed November 2017).

Recently, reports indicate that poaching of Asian elephants is occurring and may be a serious problem in some range states [11, 12, 13]. In Myanmar, for example, the government reported that 133 elephants died between 2010 and 2016, 61 from poaching [14]. The same report states that 25 elephants were killed in 2016, suggesting the rate of poaching is increasing. This is especially worrisome for Myanmar where the wild elephant population collapsed from an estimated 10,000 individuals in the 1940s, to an estimated 1,430–2,065 today [9, 15, 16, 17]. Our data are from a project focused on reducing human-elephant conflict (HEC) in Myanmar, in three relatively small areas: the southern foothills of the Bago Yoma mountain range, the Ayeyarwady Delta, and the southern reaches of Tanintharyi (Fig 1). Information on poaching was incidental to project aims, given poaching was not perceived as a problem at the onset of the work. Here, we present survival data from GPS-satellite collared elephants, and patrol and informant based information on the causes of elephant mortality collected through an associated community educational outreach program, Human-elephant Peace (H.El.P.), and by the Myanmar Ministry of Natural Resources and Environmental Conservation (MONREC) officials.

## Methods

### Study areas

In the Bago Yoma mountain range of south central Myanmar, a large portion of the elephant habitat was lost in the construction of two dams created to store water for the capital city Yangon during the dry months. Dam workers and others have since settled in the area, leading to significant HEC. Elephants were initially displaced from their habitat but quickly returned to take advantage of the crops grown by the villagers, and the abundant and stable water supply from the reservoirs. HEC in this area is the one of the highest in Myanmar, with 3–6 people killed each year and extensive crop damage, particularly of rice paddies and sugarcane plantations (MONREC, personal communication). Reports from the area indicate villagers may be assisting poachers to reduce the elephant population and prevent more conflict (Khin Maung Gyi, personal communication). We collared 15 elephants at this study site between December 2014- January 2017 and conducted community outreach programs at this site December 2016-present.

The Ayeryarwady Delta is located on the west coast of Myanmar and is dominated by mangrove and alluvial floodplain habitats. It is a major center for rubber and peppercorn production in Myanmar. Elephant habitat in this area is heavily fragmented by the expansion of agriculture, and development of major highways. Reported HEC events include increasing amounts of injuries and deaths within the local human populations, including the deaths of at least four people in 2016, as well as crop damage and loss of livestock [19]. We conducted community outreach programs beginning in December 2016 in the Pathein District which has a population of more than 1,630,000 people [20]. Elephant poaching is also prominent in this area and several poachers were arrested in this region in early August 2017 (MONREC, personal communication).

Our study site in the Tanintharyi region is on a narrow strip of land between the Andaman Sea and Thailand, and located in the southern-most region of Myanmar. This area has a mosaic of oil palm and rice plantations, and one of the largest remaining lowland evergreen rainforests in mainland Southeast Asia [21]. It has a significant amount of HEC, primarily in the agricultural fields, though the reported human injuries and loss of life are lower than in our other two areas of study. Due to its proximity to Thailand, elephants in this region may face a higher risk of live capture for the illegal wildlife trade to other parts of Asia than do elephants elsewhere in Myanmar. We are tracking elephant movement in this area, with 4 elephants collared in March 2017, and will expand the community outreach effort to this study site in late 2017.

### Identifying poaching events

We documented the loss of elephants in our study areas in three ways: loss of collared elephants, carcasses found as a result of discussions held by H.El.P with community members, and reports of carcasses found from MONREC. Our capture and collaring methods follow well-established procedures approved by the Smithsonian National Zoological Park Animal Care and Use Committee (Proposal #14–31) and are permitted through a memorandum of understanding with MONREC. All efforts were made to minimize any harm to the elephants experienced during the collaring procedure. Our research team fitted 19 elephants (16 male, 3 female) with satellite-GPS collars in four separate collaring drives: three in the Bago Yoma in December 2014, January- February 2016, and December 2016-February 2017; and one in Tanintharyi in March 2017. Elephants were captured in areas of high conflict near human habitation, including in sugarcane plantations, rice paddies and oil palm plantations. The collars record positions hourly and send a mortality signal if the collar has been immobile for more than 24 hours.

The H.El.P. community outreach team traveled to schools and community centers in areas of high HEC in the Bago Yoma and Ayeryarwady Delta. Their primary goals were to increase understanding of elephant ecology and reduce the incidence of human-elephant encounters. During these meetings, local community members alerted district leaders and the team to the sites of eight poaching events. The H.El.P. team documented the presence of any elephant carcasses reported and notified the local government to dispose of any remains.

In addition to the carcasses reported by our team, local government officials from MONREC were informed of elephant carcasses from local residents as well as encountering them on anti-poaching patrols by mahouts who form Myanmar’s Emergency Elephant Response Unit (EERU). Rangers and mahouts from the EERU are tasked with patrolling hotspots of poaching and investigating when a poaching event occurs. The Myanmar government has arrested at least 15 people on poaching charges in the past year as a result of this increased patrolling in the Bago Yoma (MONREC, personal communication). The work of the EERU contributed to the number of uncollared elephant carcasses located in the Bago Yoma, in addition to the two carcasses located in central Myanmar.

### Assessing population impacts

To estimate potential impacts of this poaching pressure on local elephant populations, we analyzed the survival of collared elephants at the Bago Yoma study site using the Kaplan-Meier product limit estimator [22]. We assumed no differences between individual animals or times of year, and accounted for censored observations when collars failed or in the single case that an individual was still active at the time of the analysis.

## Results

**Fig 2:**
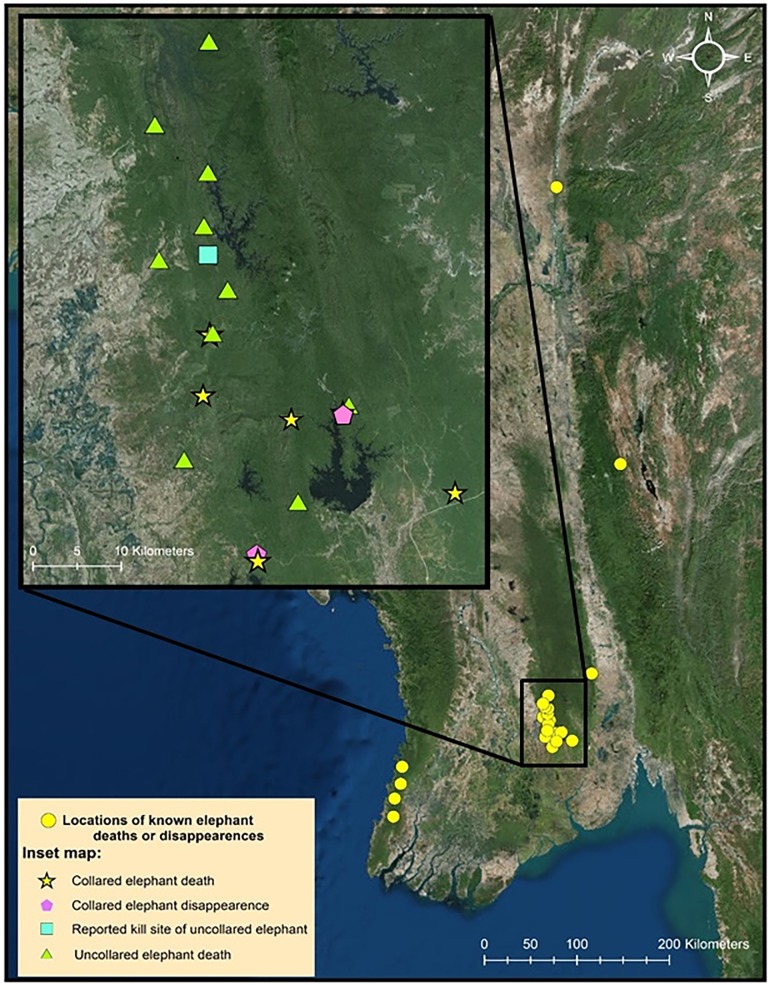
Locations of elephant deaths and disappearances in Myanmar. Inset map shows elephants lost at the Bago Yoma field site, including collared and uncollared elephants. Image source: World Imagery: Esri, DigitalGlobe, Earthstar Geographics, CNES/Airbus DS, GeoEye, USDA FSA, USGS, Getmapping, Aerogrid, IGN, IGP, and the GIS User Community (accessed November 2017).

**Fig 3:**
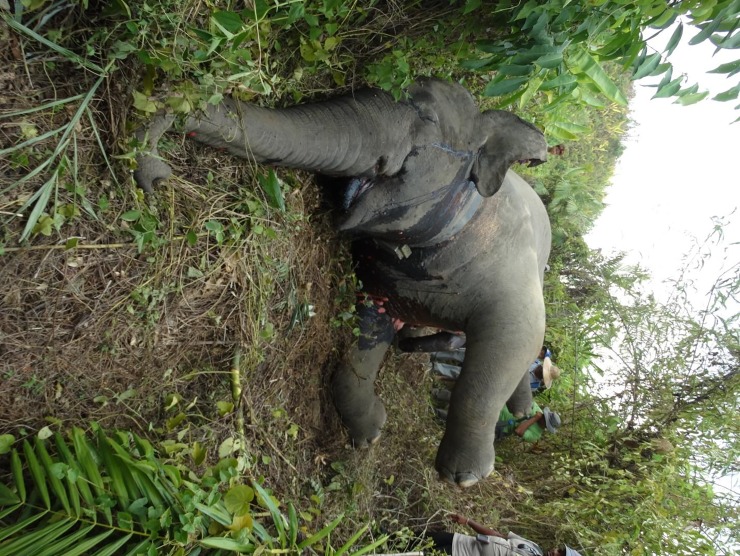
Elephant 201601. The carcass of Elephant 201601 was located on May 18^th^, 2016 in a rubber plantation. Credit: Christie Sampson.

**Fig 4:**
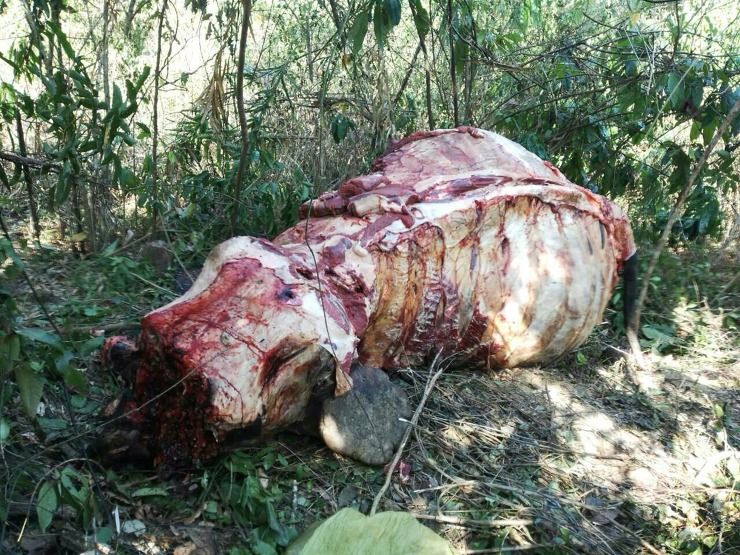
Skinned elephant cacass. The skinned carcass of an elephant found in April in Ayeyarwady. Credit: Dr. Zaw Min Oo.

**Fig 5:**
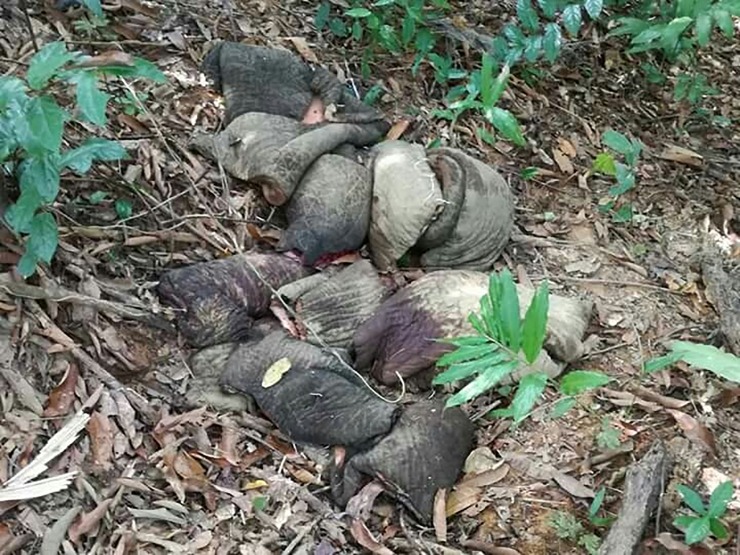
Poaching for elephant body parts. Elephant skin and trunks removed by poachers in Ayeyarwady. Credit: Dr. Zaw Min Oo.

Between March 2015 and June 2017, we found the poached carcasses of five of the 19 elephants collared in the Bago Yoma area (Table 1, Figs 2 and 3, S1 Table). During this time, two more collared elephants stopped transmitting. Movement patterns prior to their disappearance suggested they were sick or injured (movement restricted to <50 m for more than 24 hours). Attempts to locate the carcasses were unsuccessful, which combined with the movement pattern and subsequent loss of signal from the collar suggest these animals were poached or captured for the live elephant trade. All seven collared elephants that were poached or disappeared were adult males, 20–45 years old. Only one had tusks. The carcasses of 11 more uncollared elephants were also discovered in the Bago Yoma area during this time (Fig 2, S1 Table). Poachers arrested by government officials admitted to killing one additional elephant and identified the area where they left the carcass, however our team was unable to locate the remains. In March 2017, our community outreach team in the Ayeyarwady Delta region discovered the carcasses of 20 elephants at a single kill site (MONREC, personal communication, Figs 4 and 5). The team subsequently found several more kill sites including one approximately 45 km away where a group of five elephants had been poached (S1 Table).

**Table 1:** Collared elephant status.

Elephant ID	Start Date	End Date	Status	Gender	Location
201401	6-Dec-14	15-May-15	Missing (presumed dead/captured)	M	Bago
201402	11-Dec-14	20-Sep-15	Dead	M	Bago
201403	15-Dec-14	11-Mar-15	Dead	M	Bago
201404	17-Dec-14	22-Jan-15	Collar fail (reused on 201405)	M	Bago
201405	7-Feb-15	27-Jun-15	Collar fail	M	Bago
201601	4-Jan-16	17-May-16	Dead	M	Bago
201602	6-Jan-16	4-Apr-16	Collar fail	M	Bago
201603	23-Jan-16	26-Mar-17	Collar fail	M	Bago
201604	26-Jan-16	18-Feb-17	Collar fail	F	Bago
201605	1-Feb-16	17-Aug-16	Collar fail	F	Bago
201606	17-Dec-16	31-Mar-17	Dead	M	Bago
201607	21-Dec-16	5-Jun-17	Missing (presumed dead/captured)	M	Bago
201608	27-Dec-16	27-Dec-16	Collar fail	M	Bago
201701	15-Feb-17	-	Active	M	Bago
201702	27-Feb-17	6-Mar-17	Dead	M	Bago
201703	14-Mar-17	-	Active	F	Tanintharyi
201704	15-Mar-17	10-May-17	Collar fail	M	Tanintharyi
201705	16-Mar-17	-	Active	M	Tanintharyi
201706	17-Mar-17	-	Active	M	Tanintharyi

Summary of the status of the collared elephants in the two study sites, the Bago Yoma and Tanintharyi, between 2015-present. The capture date and date of last position transmission is provided for each collared elephant.

## Discussion

Our team and collaborators have identified the poaching of over 40 elephants in the localized study areas where our efforts have focused. Since the bodies were often butchered or mutilated, it was often not possible to identify the sex or age of some animals. In the Bago Yoma region, of the five carcasses of collared male elephants found, only one was a tusker. The deaths or disappearances of at least 19 adult elephants within the Bago Yoma field site in such a short time frame added to discoveries of mass killings in nearby areas indicate a critical threat to the survival of the elephant population in Myanmar. We directly attribute 12 uncollared and at least five collared elephant deaths to poaching and the illegal wildlife trade. The movement patterns and observations made by MONREC staff prior to the deaths of an additional two collared elephants suggest that they were healthy, young males unlikely to have died of natural causes.

Poisoning using darts loaded with widely available herbicides is a common method used by poachers to kill elephants (Zaw Min Oo, personal communication). The poison can take 2–3 days to take effect, which may account for the restricted movement patterns we saw in the final days of several collared elephants and explain why collared elephants located within a few days of their death were found unmutilated, as MONREC staff may have located the elephant before the poachers.

Most of the elephants killed were slaughtered for meat and skin. Given the size of the animals, the amount of material that would need to be stored and transported, and the butchering skills necessary to skin an elephant, this indicates experienced and well-coordinated poachers. According to local informants, the meat and skin are quickly moved across the border with China, indicating a connection with well-organized criminal groups and trafficking networks. This information aligns with previous studies [23] and more recent media articles showing markets in towns on the Myanmar/China border such as Mong La as hot spots for the illegal wildlife trade.

The dynamics of elephant poaching and illegal trafficking appear to have shifted from ivory and live animals to the skin and meat trade, which is spreading across the region with reports also from Northern India and Thailand [23, 24]. The markets for non-ivory elephant products are poorly understood. The main driver seems to be the use of elephant skin as a medicinal product for treating skin fungi and infections, but also intestinal disease in people [10]. The skin is ground to a powder and then frequently combined with elephant fat to produce a paste for application [10]. Elephant feet are also ground up for medicinal use or converted to furniture. Elephant skin is used in jewelry production with bracelets of cured and polished subcutaneous layer beads costing upwards of $115 USD. According to our local sources, the trade in meat for food concentrates on the trunk and genitalia.

Past poaching events that focused on ivory affected only male Asian elephants—females don’t have tusks in this species—skewing the sex ratio but often leaving enough male breeders to slow declines [25, 26]. This new crisis, with increasing demand for elephant skin for jewelry and medical products, and elephant trunks and legs for furniture, now places all elephants at risk. Considering the low reproductive rate, long gestation period and long inter-calving period of Asian elephants, the targeting of critical female breeders may have devastating consequences for the survival of Asian elephant populations.

**Fig 6:**
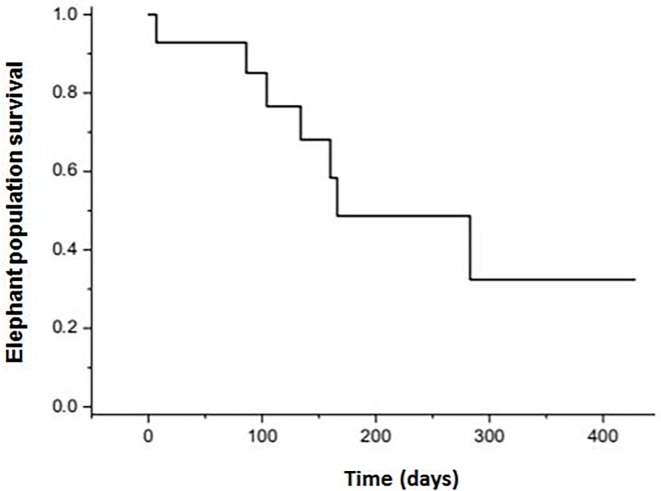
Bago Yoma elephant population survival estimation. Population survival estimation over time utilizing data from the Bago Yoma collared elephant study.

In the Bago Yoma area, six of the 14 individuals for which we had survival data were lost to follow up either due to collar failures and a seventh was still alive at the time of analysis. Only two of the individuals tested were females. Therefore, we could not measure survival differences between the sexes, age classes or seasons. We also could not assign confidence to any parametric estimate of survival, but note that the median (non-parametric) survival was 0.445 years (Fig 6). Though our sample size is small, these results clearly demonstrate that this level of poaching in unsustainable for this population.

The elephants tracked in the Tanintharyi region do not experience the same poaching pressure as the elephants in the Bago Yoma and Ayeryarwady areas. Though the sample size is smaller (n = 4), these particular animals have outlasted a majority of those in the Bago study area. Community outreach teams will expand their efforts to include sites within this region to gain a better understanding of whether the data received from our collared elephants accurately reflects the situation of poaching on the ground.

Myanmar has the largest remaining wildlands of all the Asian elephant range states [27, 28], with wide swaths of intact forest currently connected by disturbed secondary forest and agriculture [21]. These wild areas provide a refuge for several endangered species including elephants, and extend beyond the within country elephant range allowing for connectivity between elephant populations especially in northern Myanmar [15, 27]. Elephants do use disturbed areas and are drawn to the interface of agriculture and intact forest by foraging opportunities. However, use of these edge habitats by elephant populations increases the occurrence of HEC and potentially places them at a greater risk of being poached due to increased visibility and accessibility. Preservation of these wildlands offers one of the best chances for sustained Asian elephant conservation globally, as current elephant habitat throughout Asia is rapidly being fragmented and converted to human dominated landscapes [27, 28]. Poaching pressure in this critical habitat could endanger future source populations which may be necessary for reintroduction of elephants into other regions of Myanmar or throughout the Asian elephant range.

This project began with two goals: a) to track wild elephants using satellite-GPS collars to better understand when, where, how and why they come into conflict with people (Dec 2014 –present) to improve elephant management practices, and b) to use community-outreach teams to teach local people how to behave safely around elephants (November 2016-present). However, the technology and indirectly-related activities of team members led to the detection of the poaching frequency of wild elephants across the study sites. Most elephant range countries are ill-equipped to combat elephant poaching and trafficking, and may need significant support from the international community to build their enforcement capacity. Yet, there are success stories from East Africa where much has been learnt about how to develop legal frameworks and law enforcement capacity, and how to work with local communities to curb wildlife poaching and trafficking. There is an urgent need to transfer these lessons, skills, and capacities to countries with populations of Asian elephants in order to inform effective conservation policy.

## Supporting information

S1 Table Locations of elephant poaching events March 2015-August 2017. A summary of the elephant deaths and disappearance documented between March 2015 and August 2017. Collared elephants refer to the elephants that were being tracked by the research team prior to their death or disappearance. Uncollared elephants were not part of the movement study, and were found either incidentally or after the research team and collaborators began searching for evidence of poaching. The location of each carcass or last position before the collar stopped transmitting is listed along with the number of individuals found at each point. The “*” indicates the two elephants, one collared and one uncollared, that were found at the same location at the same time.
(DOCX)
