# Supplementary material for: New elephant crisis in Asia—Early warning signs from Myanmar
Source: PLoS One. 2018 Mar 13;13(3):e0194113. doi: 10.1371/journal.pone.0194113 (PMC5849331; doi:10.1371/journal.pone.0194113)
Supplement: S1 Table — A summary of the elephant deaths and disappearance documented between March 2015 and August 2017. Collared elephants refer to the elephants that were being tracked by the research team prior to their death or disappearance. Uncollared elephants were not part of the movement study, and were found either incidentally or after the research team and collaborators began searching for evidence of poaching. The location of each carcass or last position before the collar stopped transmitting is listed along with the number of individuals found at each point. The “*” indicates the two elephants, one collared and one uncollared, that were found at the same location at the same time. (DOCX) [file pone.0194113.s001.docx]

**S1 Table. Locations of elephant poaching events March 2015-August 2017.** A summary of the elephant deaths and disappearance documented between March 2015 and August 2017. Collared elephants refer to the elephants that were being tracked by the research team prior to their death or disappearance. Uncollared elephants were not part of the movement study, and were found either incidentally or after the research team and collaborators began searching for evidence of poaching. The location of each carcass or last position before the collar stopped transmitting is listed along with the number of individuals found at each point. The “*” indicates the two elephants, one collared and one uncollared, that were found at the same location at the same time.

| **Elephant Status** | **Easting** | **Northing** | **Notes** |
| --- | --- | --- | --- |
| Collared | 96 15.163744 | 17 22.357231 | Single carcass |
| Collared | 96 3.2923 | 17 18.608298 | Missing |
| Collared | 96 5.551078 | 17 26.553063 | Single carcass |
| Collared | 96 0.416285 | 17 31.529855 | *Two carcasses |
| Collared | 96 3.427236 | 17 18.045972 | Single carcass |
| Collared | 96 8.368966 | 17 26.649866 | Missing |
| Collared | 96 0.161103 | 17 27.87836 | Single carcass |
| Uncollared | 95 57.294987 | 17 35.869689 | Single carcass |
| Uncollared | 96 1.678012 | 17 34.161159 | Single carcass |
| Uncollared | 96 0.804691 | 17 31.635474 | *Two carcasses |
| Uncollared | 96 0.180836 | 17 38.107619 | Single carcass |
| Uncollared | 96 9.242538 | 17 27.637522 | Two carcasses |
| Uncollared | 96 0.255452 | 17 41.277522 | Single carcass |
| Uncollared | 95 58.336526 | 17 41.979354 | Single carcass |
| Uncollared | 96 4.222107 | 17 47.553245 | Single carcass |
| Uncollared | 95 59.029788 | 17 24.051636 | Single carcass |
| Uncollared | 96 6.039481 | 17 21.375014 | Single carcass |
| Uncollared | 94 27.358324 | 16 43.321523 | Three carcasses |
| Uncollared | 94 24.308185 | 16 24.922116 | Six carcasses |
| Uncollared | 94 32.510381 | 16 53.517996 | Twenty carcasses |
| Uncollared | 94 32.87059 | 17 5.137739 | Single carcass |
| Uncollared | 96 29.2785 | 17 59.561469 | Five carcasses |
| Uncollared | 96 43.18588 | 20 8.697419 | Single carcass |
| Uncollared | 96 11.762913 | 22 46.873153 | Single carcass |
| Uncollared | 96 0.590197 | 17 36.407565 | Reported kill site |
